# Effectiveness and safety of transvenous extraction of single- versus dual-coil implantable cardioverter-defibrillator leads at single-center experience: Erratum

**DOI:** 10.1097/MD.0000000000018979

**Published:** 2020-01-17

In the article, “Effectiveness and safety of transvenous extraction of single- versus dual-coil implantable cardioverter-defibrillator leads at single-center experience”,^[1]^ which appears in Volume 98, Issue 30 of *Medicine*, the last line of Table 2 appears incorrectly as “During 30-d postoperative period procedure-related, n (%)” and should be “During 30-d postoperative period nonprocedure-related, n (%)”.
